# Prevalence of common mental disorders and associated factors among pregnant women attending antenatal care at the University of Gondar Comprehensive Specialized Hospital, Northwest Ethiopia, 2023

**DOI:** 10.3389/fpsyt.2025.1544254

**Published:** 2025-08-04

**Authors:** Kiber Temesgen, Berhanie Getnet, Biksegn Asrat, Agegnehu Amare

**Affiliations:** ^1^ Department of Psychiatry, Mizan-Tepi University, Mizan-Aman, Ethiopia; ^2^ Department of Psychiatry, College Of Medicine and Health Science, University of Gondar, Gondar, Ethiopia

**Keywords:** common mental disorder, pregnant women, Northwest, Gondar, Ethiopia

## Abstract

**Background:**

Common mental disorders (CMDs) such as depression and anxiety are prevalent during pregnancy. CMDs are public health concerns because of the implications for the health of both the mother and the fetus. This study aimed to determine the prevalence of CMDs and associated factors among pregnant women attending antenatal care at the University of Gondar Comprehensive Specialized Hospital (UoG CSH), Northwest Ethiopia.

**Methods:**

An institution-based cross-sectional study was conducted from 26 September to 28 October 2023 among pregnant women attending antenatal care at the UoG CSH. Study participants were selected using a systematic random sampling technique. Data were collected using pre-designed tools like the Self-Reporting Questionnaire (SRQ-20) and the Oslo-3 Social Support Scale through face-to-face interviews. The collected data were entered into Epi data version 4.6.02 and analyzed using STATA version 14. Bivariable and multivariable logistic regression were used to identify factors associated with CMDs.

**Results:**

Of the 407 pregnant women, 170 (41.8%) fulfilled the criteria for CMDs. In the multivariable analysis, financial instability (AOR = 1.66, 95% CI: 1.02, 2.69), poor social support (AOR = 2.60, 95% CI: 1.41, 4.81), emotional or physical abuse (AOR = 3.86, 95% CI: 1.79, 8.30), history of mental illness (AOR = 4.00, 95% CI: 1.24, 12.86), and unwanted pregnancy (AOR = 3.02, 95% CI: 1.02, 8.94) were significantly associated with CMDs.

**Conclusion and recommendation:**

This study indicated that the prevalence of CMDs was high among pregnant women attending antenatal care at the UoG CSH. Those who had financial instability, poor social support, emotional or physical abuse, history of mental illness, and unwanted pregnancy were prone to CMDs. Therefore, early screening and monitoring of CMDs among pregnant women are important to reduce possible negative impacts on the health of women.

## Introduction

Common mental disorders (CMDs) are non-psychotic mental disorders (1) that manifest as depression, anxiety, and somatic disorders that compromise day-to-day functioning (2–4). CMDs have multiple implications that change thoughts, feelings, behavior, and interpersonal relationships (5), characterized by symptoms such as insomnia, fatigue, irritability, difficulty in concentrating, and somatic complaints (6–8).

CMDs contribute to a high proportion of disability in the global burden of disease (9, 10). It accounts for 14% of the total disease burden globally and is one of many public health challenges today (11–13). CMDs among pregnant women are a global concern (14, 15) because of the load and restrictions on participation in daily activities, as well as the risk to the growing fetus's neurocognitive development and unfavorable pregnancy outcomes (16–19).

Although economic conditions make women more vulnerable in low- and middle-income countries (LMICs), 2012 research comparing maternal CMDs to neonatal and obstetric outcomes found antenatal CMDs ranged from 12% to 43% throughout pregnancy in both high-income and low-income countries (20). In antenatal clinics in LMICs, one in six pregnant women suffers from CMDs, particularly depression and anxiety (19). In sub-Saharan Africa, the prevalence of CMDs ranges from 8.3% to 41% during pregnancy (21–23).

Ethiopia is one of the low-income countries with high rates of CMDs in pregnant women ranging from 12% to 46.6%, according to the evidence reported from studies conducted in different areas of the country (16, 24–27).

**Figure 1:**
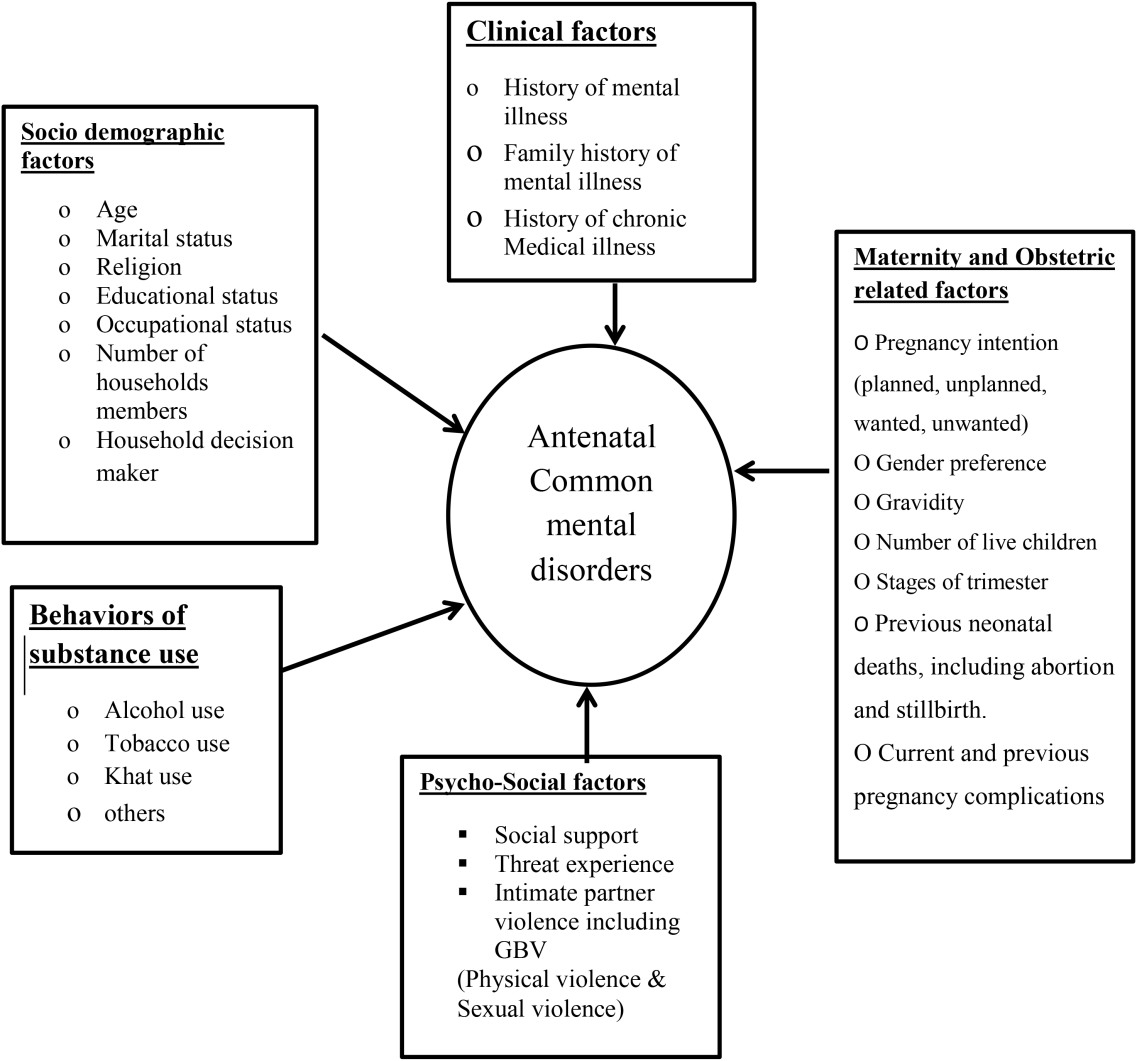
Conceptual framework representing CMDs and their associated factors among pregnant women, 2023, adopted from reviewing the literature.

Numerous independent research have shown that antenatal CMDs are influenced by intimate partner abuse, low socioeconomic level, unplanned pregnancy, lack of social support (11, 16, 27), and complications in past or index pregnancy (8). Additionally, poor health condition prior to conception, such as headache, diabetes mellitus, hypertension (26), illiteracy, and being single, widowed, divorced, or separated are associated with antenatal CMDs (**Figure 1**) (4).

Women who have CMDs throughout pregnancy are less likely to seek prenatal care, and they may also have lower weight gain during pregnancy (28), which increases the chance of complicated delivery and poor child health outcomes such as stillbirth, low birth weight, prematurity, and neonatal mortality and morbidity (3, 29–32).

Untreated CMDs during the antenatal period may continue in the postnatal period, thus resulting in decreased emotional involvement and hostility toward the newborn (24, 33–36).

CMDs are serious health problems, but are a neglected component of care for women during pregnancy (33). If left untreated, both short- and long-term physical, social, and occupational disabilities are inevitable (37). The long-term effects of CMDs are due to the relapsing nature of the problem, poor adherence, and treatment-seeking behavior (38). CMDs have an effect on healthcare expenses and economic productivity, and they are linked to a poor prognosis of comorbid diseases (12).

Despite the high prevalence and negative outcomes that CMDs have among pregnant women, there are limited studies in LMICs, including Ethiopia. Ethiopia is a country with a wide range of sociocultural characteristics, and the factors that contribute to CMD might vary from culture to culture even within the research that is already available. Therefore, the findings of this study provide information related to the prevalence of antenatal CMDs and their associated factors in Northwest Ethiopia. Thus, the evidence is important for researchers as an initial input for their further investigation.

## Materials and methods

### Study design, period, and setting

An institution-based cross-sectional study was conducted among pregnant women attending ANC service at the University of Gondar Comprehensive Specialized Hospital (UoG CSH) from 26 September to 28 October 2023, Gondar is the capital city of the Central Gondar zone, which is located Northwest of Addis Ababa. It is located at a distance of 738 km from Addis Ababa, the capital city of Ethiopia, and at a distance of 186 km away from Bahir Dar, the capital city of Amhara regional state. The total projected Gondar population was 323,900. Gondar town has one comprehensive, specialized hospital, one general hospital, eight public health centers, and 14 health posts.

### Source population

All pregnant women who visited the UoG CSH made up the source population.

#### Study population

All pregnant women who visited the UoG CSH and who were presented at the time of data collection comprised the study population. Pregnant women who could not communicate and who were acutely sick during data collection were excluded from the study.

### Sample size and sampling procedure

The sample size was determined by using the single-population proportion formula based on the assumption of 46.6% prevalence of CMDs from a study conducted in Ethiopia (27) and 1.96 *Z* value of standard normal distribution. By using 95% confidence interval (CI) and 5% margin of error and by adding 10% non-response, the final sample size was 419. To choose study participants, a methodical random sampling technique was used. By dividing the total number of pregnant women who had visited the antenatal clinics at the UoG CSH by the sample size, the sampling interval was calculated. A formula of *k* = *N*/*n* was used to get an interval of 3 (*k* = 1,504/419 ≈ 3), so the participants were selected every three intervals. The lottery method was used to choose the first person. If the selected individual is not present or refused to participate, the data collectors substituted the next participant and continued the process starting from the interviewed participant based on the interval; hence, selected participants were interviewed by the data collector at regular intervals from among consecutive women visitors attending antenatal care.

### Data collection procedures and instruments

Data were collected from the study subjects through face-to-face interviews using structured questionnaires that consisted of items on socio-demographic, substance use, clinical, psychosocial, and maternity- and obstetrics-related characteristics. Participants were approached in their waiting rooms and interviewed individually to ensure privacy. CMDs were assessed by the Self-Reporting Questionnaire (SRQ-20), which was designed by WHO for developing countries. It was translated into Amharic language, tested and validated in Ethiopia. Each of the 20 questions was scored 0 (no) or 1 (yes), with the total score ranging from 0 to 20; a cutoff score (SRQ-20 ≥ 7) was used to determine probable cases of mental disorder or poor mental health (16). Stressful life events were assessed by a List of Threatening Experiences questionnaire (LTE-Q), which has good test–retest reliability (Kappa: 0.61–0.87) (39) and predictive validity in Debre-Tabor (40). Social support was assessed using the Oslo-3 Social Support Scale (OSS-3) (41). The presence of victimization or intimate partner violence during pregnancy was obtained by Abuse Assessment Screening (AAS), which was developed by the Nursing Research Consortium on Violence (27). To examine substance use history, respondents were asked: "Have you ever used any substance in the last three months or in your lifetime?" and the responses were Yes/No (11).

The pre-coded structured questionnaire included self-report based on clinical and psychiatric history and previous and current obstetric history after an extensive literature review (16).

### Study variables

#### Dependent variable

The study's dependent variable was common mental disorder (CMD) = yes/no.

#### Independent variables

The study's independent variables were socio-demographic factors, which included age, marital status, religion, educational status, occupational status, number of household members, and household decision-maker; the other independent variables were psychosocial factors, which included stressful life events, social support, and intimate partner violence (physical violence and sexual violence). Factors like history of mental illness, history of chronic medical illness, and family history of mental illness were included under the category of clinical factors. The remaining independent variables were substance and related factors, which include alcohol drinking, cigarette smoking, and khat chewing, and maternity- and obstetrics-related factors, which include pregnancy intention, gender preference, gravidity, number of live children, stages of trimester, previous neonatal deaths including abortion, current and previous pregnancy complications, and history of gynecological operation.

### Operational definitions of key concepts

Common mental disorder (CMD); A probable case of CMD is defined in the current study for those participants who scored based on a cutoff point greater than or equal to seven during SRQ-20 screening (9, 23).

History of mental illness: This refers to previous diagnosed psychopathology for participants having at least one or more known self-reported mental illnesses before the study period.

Chronic medical illness: This refers to a condition when subjects have at least one or more known self-reported chronic diseases before the study period (16).

Stressful life events: The presence of specific life events explained by experience of one or more adverse life events in the previous 6 months, assessed using the 12-item List of Threatening Experiences (LTE) (11, 39).

Social support: Assessed using OSS-3 and scored as "poor support" (3–8), "moderate support" (9–11), and "strong support" (12–14) (41).

Substance use: Ever used substance (alcohol, cigarette, and khat) in one's lifetime and current substance use (alcohol, cigarette, and khat) in the past 3 months (26).

Intimate partner violence: A "yes" response to any question of AAS for pregnancy is considered positive for partner violence (42).

### Data quality control

Standardized questionnaires were used, which were translated into Amharic and translated back to English to check for consistency and understandability of the tool. Four BSc midwives were recruited for data collection. A training about the content of questionnaires for data collectors and supervisors was given. A pre-test was conducted 2 weeks before the actual data collection on 5% (*n* = 21) of the sample size at Ayira General Hospital. Supervision by the two PhD qualified supervisors and a PI was carried out. During data collection, filled questionnaires were checked for completeness and consistency.

### Data analysis procedure

Data were coded, entered, and cleaned using Epi data version 4.6.02 and then they were exported to STATA version 14. Then, the data were analyzed to generate descriptive statistics: means, frequency, percentages, and standard deviations. In order to determine the association between dependent and independent variables, adjusted odds ratio was employed using logistic regression and the significance level was determined using a CI of 95%. Bivariable analysis was carried out to assess the association of each independent variable with the dependent variable.

## Results

### Socio-demographic data of respondents

From 419 approached participants, 407 consented to take part and completed the interview in the study, yielding a response rate of 97.1%. Among the study participants, 33.9% were between the ages of 25 and 29 years with a mean of 28.6 years (SD ±5.7). The majority of the study participants (95.8%) were married in their current relationship, and 90.4% were Orthodox Christian followers. Approximately 25.6% of the participants completed secondary level of education and 37.6% were housewives (**Table 1**).

**Table 1:** Socio-demographic information of pregnant women in UoG CSH, Gondar, Northwest Ethiopia, 2023 (*n* = 407).

Variable	Categories	Frequency	Percentage
Age	18–24	99	24.3
	25–29	138	33.9
	30–34	101	24.8
	≥35	69	17.0
Marital status	Married	390	95.8
	Single/divorced/widowed	17	4.2
Religion	Orthodox	368	90.4
	Muslim	29	7.1
	Others*	10	2.5
Level of education	Illiterate	50	12.3
	Primary level education (1-8)	68	16.7
	Secondary level education (9-12)	104	25.6
	Diploma	99	24.3
	Degree and above	86	21.1
Occupation	Housewife	153	37.6
	Civil servant	89	21.9
	Private servant	74	18.2
	Merchant/private business	53	13.0
	Others**	38	9.3
Number of households	0–2	80	19.7
	3–4	246	60.4
	≥5	81	19.9
Household decision-maker	Self	20	4.9
	Husband	47	11.5
	Together	340	83.6

*Bete-isral, Protestant, Catholic. **Student, day laborer, unemployed.

### Substance use-related factors

Findings suggest that almost 70.0% of women used alcoholic beverages like traditional alcoholic drinks [local "areke", "tella", and "tej" (honey wine)] or industrial beverages like wine and beer at least once in their lifetime. Among these, 21.1% were consuming alcoholic beverages during the study period. From the total number of study participants, 4.9% reported ever chewing khat in their lifetime, whereas 1% was consuming khat during the study period. Approximately 1.2% of the respondents had ever used tobacco in their lifetime, and the prevalence of tobacco use during the study period was not statistically significant.

### Psychosocial factors

Among the respondents, 15.2% had (LTE) health risks, 10.3% had lost a loved one, 34.6% had financial instability, 17.2% had relationship problems, and 9.3% had legal problems. From study participants, 11.8% reported that they had been emotionally or physically abused. Approximately 8.6% of participants reported being forced to have sexual intercourse within the last year. Of the participants, approximately 31.7% had poor social support, whereas 45.7% and 22.6% had moderate and strong social support, respectively (**Table 2**).

**Table 2:** Psychosocial characteristics of pregnant women in UoG CSH, Gondar, Northwest Ethiopia, 2023 (*n* = 407).

Variable	Categories	Frequency	Percentage
Categories of stressors (LTE) Health risk	Yes	62	15.2
	No	345	84.8
Loss of loved one	Yes	42	10.3
	No	365	89.7
Financial instability	Yes	141	34.6
	No	266	65.4
Relationship problem	Yes	70	17.2
	No	337	82.8
Legal issue	Yes	38	9.3
	No	369	90.7
History of emotional or physical abuse	Yes	48	11.8
	No	359	88.2
Forced sexual activities in last one year	Yes	35	8.6
	No	372	91.4
Social support	Poor	129	31.7
	Moderate	186	45.7
	Strong	92	22.6

### Clinical factors

**Figure 2:**
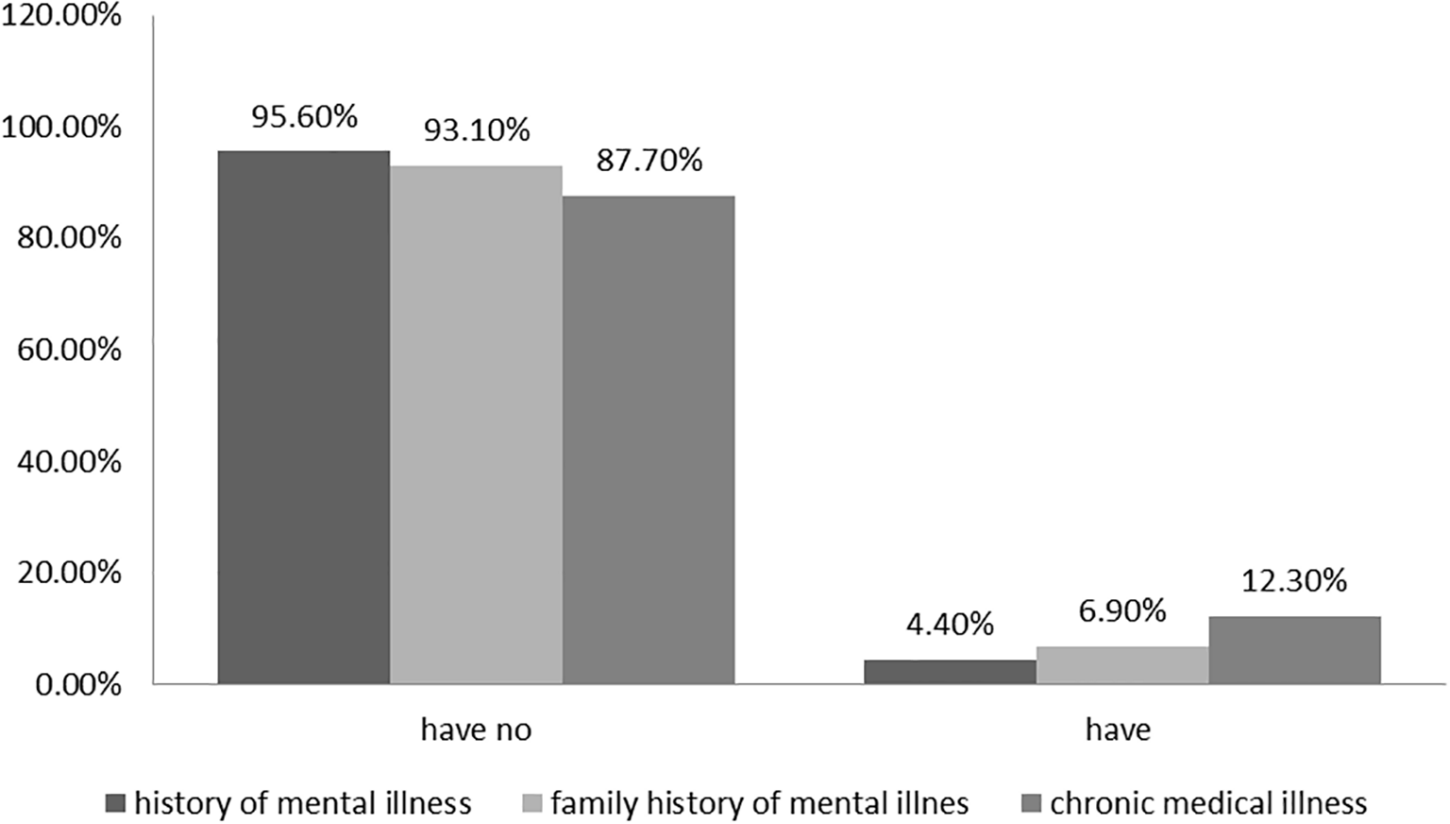
Clinical factors of pregnant women (*n* = 407) in UoG CSH, Gondar, Northwest Ethiopia, 2023.

Participants were asked about various clinical conditions and 12.3% reported having a history of chronic medical illness, and 4.4% had a history of mental illness. Additionally, 6.9% had a family history of mental illness (**Figure 2**).

### Maternity- and obstetrics-related factors

The majority of the study participants (42.0%) were in the third trimester of their pregnancy, and 58.9% had been pregnant two to four times before. In this sample, 4.9% had an unwanted pregnancy, 7.9% had a history of abortion, and 4.4% had a prior history of neonatal death. More than half (53.6%) of the participants preferred to have a male child, and 42.2% of the participants had one to two children. From the total respondents, 10.1% had a history of previous pregnancy-related complications, and 11.1% had a history of current pregnancy complications. Approximately 16.2% of the participants had a history of a gynecological operation (**Table 3**).

**Table 3:** Maternity and obstetric related factors of pregnant women in UoG CSH, Gondar, Northwest Ethiopia, 2023 (*n* = 407).

Variable	Categories	Frequency	Percentage
Pregnancy intention	Planed/wanted	282	69.3
	Unplanned but wanted	105	25.8
	Unwanted	20	4.9
Gestational age	First trimester	87	21.4
	Second trimester	149	36.6
	Third trimester	171	42.0
Gender preference of child	Looking for male	218	53.6
	Looking for female	189	46.4
Gravidity	One	117	28.8
	Two to four	240	58.9
	Five and above	50	12.3
Number of live children	None	122	30.0
	One to two	172	42.2
	Three and above	113	27.8
History of past pregnancy complication	Yes	41	10.1
	No	366	89.9
Current high-risk pregnancy	Yes	45	11.1
	No	362	88.9
History of neonatal death	Yes	18	4.4
	No	389	95.6
History of abortion	Yes	32	7.9
	No	375	92.1
History of gynecological operation	Yes	66	16.2
	No	341	83.8

### Prevalence of common mental disorders and associated factors

In this study, the overall prevalence of CMD among pregnant women was 41.8% (95% CI: 37.0, 46.6). Approximately 21.6% of the women had no CMD symptoms and 36.6% had low CMD symptoms. The most common symptoms reported were tiredness (34.2%), headaches (33.2%), poor appetite (31.7%), and sleep disturbance (30.5%).

Relationship problems, financial instability, poor social support, emotional or physical abuse, forced sexual activities in the last year, current alcohol use, history of mental illness, family history of mental illness, unwanted pregnancy, complications with current pregnancy, history of neonatal death, and gynecological operation were factors associated with CMDs at *p* < 0.25 in binary logistic regression.

Finally, the multivariable analysis model indicated that having financial instability, poor social support, history of emotional or physical abuse, history of mental illness, and unwanted pregnancy were found to be significantly associated with CMDs with 95% CI and at *p* < 0.05.

Pregnant women with financial instability had 1.66 times (AOR = 1.66, 95% CI: 1.02, 2.69, *p* = 0.04] higher odds of having CMDs than women with adequate and stable financial access.

Pregnant women with physical or emotional abuse had 3.86 times (AOR = 3.86, 95% CI: 1.80, 8.31, *p* = 0.001) higher odds of having CMDs than women who had not been abused.

Pregnant women with poor social support had 2.6 times (AOR = 2.60, 95% CI: 1.41, 4.81, *p* = 0.002) higher odds of having CMDs than women who had strong social support.

Pregnant women with a past history of mental illness had four times (AOR = 4.00, 95% CI: 1.25, 12.87, *p* = 0.02) higher odds of having antenatal CMDs as compared to those who had no history of past mental illness.

Women with unwanted pregnancies had nearly three times (AOR = 3.03, 95% CI: 1.02, 8.95, *p* = 0.04) higher odds of having CMDs as compared to women with planned/wanted pregnancies (**Table 4**).

**Table 4:** Bivariable and multivariable analysis of factors associated with common mental disorders among pregnant women in UoG CSH, 2023 (*n* = 407).

Variables	Categories	CMD		COR (95%CI)	AOR (95%CI)	*P*-value
		Yes	No			
Relationship problem	Yes	37	33	1.72 (1.03, 2.89)	1.09 (0.59, 2.04)	0.77
	No	133	204	1	1	
Financial instability	Yes	76	65	2.14 (1.41, 3.24)	1.66 (1.02, 2.69)^*^	0.04
	No	94	172	1	1	
Social support	Poor	75	54	2.73 (1.57, 4.77)	2.60 (1.41, 4.81)^**^	0.002
	moderate	64	122	1.03 (0.61, 1.74)	0.82 (0.45, 1.48)	0.52
	Strong	31	61	1	1	
History of emotional or physical abuse	Yes	36	12	5.04 (2.53, 10.02)	3.86 (1.80, 8.31)^***^	0.001
	No	134	225	1	1	
Forced sexual activities in last one year	Yes	20	14	2.12 (1.04, 4.34)	2.18 (0.97, 4.90)	0.06
	No	150	223	1	1	
Current alcohol use	Yes	45	41	1.72 (1.07, 2.78)	1.48 (0.83, 2.65)	0.18
	No	125	196	1	1	
History of psychiatric illness	Yes	13	5	3.84 (1.34, 10.99)	4.00 (1.25, 12.87)^*^	0.02
	No	157	232	1	1	
Family history of psychiatric illness	Yes	17	11	2.28 (1.04, 5.01)	1.48 (0.63, 3.50)	0.37
	No	153	226	1	1	
Pregnancy intention	Unwanted	14	6	3.44 (1.28, 9.21)	3.03 (1.02, 8.95)^*^	0.04
	Unplanned but wanted	42	63	0.98 (0.62, 1.55)	1.04 (0.61, 1.75)	0.88
	Planned/wanted	114	168	0	0	
Complication with current pregnancy	Yes	25	20	1.87 (1.00, 3.49)	1.65 (0.80, 3.41)	0.17
	No	145	217	1	1	
History of neonatal death	Yes	12	6	2.92 (1.07, 7.95)	2.05 (0.69, 6.09)	0.19
	No	158	231	1	1	
History of gynecological operation	Yes	35	31	1.72 (1.01, 2.92)	1.67 (0.91, 3.05)	0.09
	No	135	206	1	1	

NB: Written in bold indicates significant association: *p-value ≤ 0.05, **p-value ≤ 0.01, ***p-value ≤ 0.001.

## Discussion

This study showed that there was a high prevalence of antenatal CMDs among pregnant women who have antenatal care follow-up at UoG CSH. The prevalence of CMDs among pregnant women in this study was 41.8% (95% CI: 37.0, 46.6), which was in line with previous studies done in Debre Birhan (45.2%) (16), Hawassa (46.6%) (27), Cameroon (42%) (23), and northeastern Brazil (43.1%) (43).

However, the prevalence of CMDs in this study was higher than findings conducted in various regions of Ethiopia, such as in Butajira and Bale zone, 12% and 35.8%, respectively (24, 26). The reason for higher prevalence of CMDs in the current study might be due to the differences in study year. Our study was conducted in 2023 while the Butajira and Bale zone studies were conducted in 2010 and 2017, respectively. The other reason was the area where the study was conducted; our study was conducted at an institution, but the Butajira and Bale zone studies were conducted at a community level.

The results were also higher than previous findings in Brazil (20.2%, 12.9%, and 20%) (20, 44, 45), Vietnam (29.9% and 37.4%) (3, 46), and the United Kingdom (24.69%) (47). The reason for the higher prevalence of CMDs in the current study might also be due to sociocultural variations, socioeconomic factors, and the availability of health facilities and health professionals between those countries and Ethiopia including factors like financial instability and other mental health risk factors such as CMDs (whether they have been detected and treated early). Another reason for this difference might be due to the differences in measurement tools. For example, Vietnam used EDS, which did not include somatic symptoms that are normative in pregnancy (3).

The current prevalence of antenatal CMDs was also lower than the previous studies done in midwestern Brazil (57.1%). This variation may be due to the level of knowledge and understanding of the participants that leads to increased health facility visits. Differences within the study population were another factor. This study included all pregnant women, but midwestern Brazil included pregnant women who had consultations that were at higher risk for CMDs (8).

The findings of this study showed that financial instability had a statistically significant association with CMDs. Pregnant women with financial instability had 1.66 times (AOR = 1.66, 95% CI: 1.02, 2.69) higher odds of having CMDs than women with adequate and stable financial access. Financial instability emerged as a stressor that had both direct and indirect effects on antenatal CMDs (26). Women were preoccupied by unpaid home duties, with complete financial reliance on their partners; thus, a failure to provide such support could pose a significant threat to maternal psychological health (48, 49). This study agreed with studies conducted in Bale, Ethiopia, and Brazil (26, 44).

The current study found that intimate partner violence had a statistically significant association with CMDs. Pregnant women with physical or emotional abuse had 3.86 times (AOR = 3.86, 95% CI: 1.80, 8.31) higher odds of having CMDs than women who had not been abused. Physically abused women might face emotional instability and inability to trust their partner, which can lead to lifelong consequences, including low self-esteem, depression, and relationship difficulties (2, 33, 50, 51).

Social support was significantly associated with CMDs. Pregnant women with poor social support had 2.6 times (AOR = 2.60, 95% CI: 1.41, 4.81) higher odds of having CMDs than women who had strong social support. In fact, having poor social interaction is prone to mental disorders, because social support is the most important mechanism to release day-to-day stress and strengthen one's coping capabilities (8). Women with poor social support might have feelings of being neglected, and not wanted, which leads participants to become socially isolated and experience emotional disturbances (52, 53). Previous studies done in Brazil and Pakistan supported this finding (44, 54).

Having a previous history of mental illness was significantly associated with antenatal CMDs. Pregnant women with a past history of mental illness had four times (AOR = 4.00, 95% CI: 1.25, 12.87) higher odds of having antenatal CMDs as compared to those who had no history of past mental illness. This might be because pregnant women were more biologically vulnerable to CMDs, and in addition, this hormonal change during pregnancy might precipitate previous mental illness or their psycho-social context may make them vulnerable to recurrent CMDs (2, 36, 44).

Regarding the intention of pregnancy by women, having unwanted pregnancy was associated with antenatal CMDs. Women with unwanted pregnancies had nearly three times (AOR = 3.03, 95% CI: 1.02, 8.95) higher odds of having CMDs as compared to women with planned/wanted pregnancies. This was possible because pregnancy causes physical, psychological, and hormonal changes (24), and this is the most likely reason why pregnancy needs physical, psychological, and financial preparation (43, 55). A previous study done in Debre Birhan showed such an association between unwanted pregnancy and CMDs during pregnancy (16).

## Limitation of the study

The major limitation of the current study is the fact that it is a cross-sectional study, and thus, the cause-and-effect relationship was difficult to establish. In addition, the data collection method used (face-to-face interview) might lead women to respond in socially acceptable ways during the process, especially in cases of substance-related questions, history of abortion, physical abuse by their spouse, and marital relationship. Social desirability bias may be more likely to occur when such delicate questions are asked.

## Conclusion and recommendations

This study revealed that the prevalence of CMDs in Ethiopia was found to be high. Financial instability, poor social support, physical or emotional abuse, history of mental illness, and unwanted pregnancy were significantly associated with CMDs among pregnant women. Mechanisms that facilitate the link between antenatal care units and psychiatric care units should be designed. Further longitudinal and robust studies with a larger sample size should be conducted to determine the risk factors and causal pathways for the occurrence of antenatal CMDs.

## Data Availability

The original contributions presented in the study are included in the article/**Supplementary Material**. Further inquiries can be directed to the corresponding author.
