# Supplementary material for: Prevalence of common mental disorders and associated factors among pregnant women attending antenatal care at the University of Gondar Comprehensive Specialized Hospital, Northwest Ethiopia, 2023
Source: Front Psychiatry. 2025 Aug 4;16:1544254. doi: 10.3389/fpsyt.2025.1544254 (PMC12358358; doi:10.3389/fpsyt.2025.1544254)
Supplement: Supplementary file 1 [file Supplementaryfile1.pdf]

Questionnaire number _________

1. **English Version Consent Form**

Dear Participants:

Good morning/afternoon? we have conducting a survey on prevalence of common mental disorders and associated factors among pregnant women. This letter serves to ask consent from you to take part in this research. Studies in other countries show different pregnant women have suffered from common mental disorders and resulted psychological or physical problem missed opportunities for psychiatric and psychological interventions. The purpose of this study is to assess prevalence of CMDs and associated factors among pregnant women attending antenatal care at the University of Gondar Comprehensive Specialized Hospital. Your participation in this research is voluntary and it is very important for achievement of the study and for paving the way for the integration of mental health service in the prevention of CMD. There is no any risk that will come to you because of your participation in this study. All the responses given by you and results obtained will be kept confidential using coding system whereby no one will have access to your response. You are not expected to give your name or phone number. Without permission from you and legal body, any part of this study will not be disclosed to third person. You have full right to refuse and withdrawal to participate in this study if you don't wish. The questionnaire will take about 30 minutes only. If you are willing to participate in this study, you need to understand and sign the agreement form, and then you can answer the possible response by yourself or will be asked to give your responses by data collectors.

Are you voluntary to fill this questionnaire? Yes ______No______

Written Consent form

I hereby confirm that I understand the contents of this document and the nature of the research project, and I consent to participate voluntarily in the research project. I understand that I am at autonomy to withdraw from the project at any time.

Signature of participant ________Date ________________________
Name and signature of data collector ______________________Date_________
Name and signature of Supervisor ________________________Date_______

2. Data collection instruments

Questionnaire on the assessment of the prevalence of CMDs and associated factors among pregnant women attending antenatal care at the university of Gondar comprehensive specialized hospital

INTRODUCTION: Thank you for agreeing to take part in this brief interview. This study is planned to assess the problem in our country above all aiming to know the prevalence of common mental disorders and associated factor among pregnant women for the integration of mental health service to regarding to common mental disorders prevention. All data obtained from you will be kept confidential. Without permission from you and the legal body, any part of this study will not be disclosed to the third person.

INSTRUCTION: The questionnaire has eight parts. It will take about 30 minutes to complete the interview. Please try to respond to all the questions. Thank you very much for your patience.

Section I: **socio-demographic information**

No	Questionnaires	Alternative response	Coding
SDQ-1	How old are you?	Age in years--------------	
SDQ-2	What is your marital status?	1. Single 3. Divorced<br>2. Married 4. Widowed	
SDQ-3	What is your religion?	1. Orthodox 3. Protestant<br>2. Muslim 4. catholic<br>5. Others-----------	
SDQ-4	What is the level of your education?	1. No formal education 4. Diploma<br>2. 1-8 5. Degree and above<br>3. 9-12	
SDQ-5	Occupational status	1. Housewife 4. Private business<br>2. Civil servant 5. other----------	

		3. Private employed	
SDQ-6	Household decision maker	1. Self 2 husband 3. Together 4. in-laws	
SDQ-7	With whom do you live?	1. With my husband 3. With my family of birth<br>2. With my husband family 4. Others	
SDQ-8	What is your average monthly income?	______________ETB birr	

Section II: List of Threatening Experiences (LTE)

S. no	List of Threatening Experiences	Response	Coding
LTEQ-9	You yourself suffered a serious illness, injury or an assault	1. Yes 2. No	
LTEQ-10	A serious illness, injury or assault happened to a close relative	1. Yes 2. No	
LTEQ-11	Your parent, child or spouse died	1. Yes 2. No	
LTEQ-12	A close family friend or another relative (aunt, cousin, grandparent) died	1. Yes 2. No	
LTEQ-13	You had a separation due to marital difficulties	1. Yes 2. No	
LTEQ-14	You broke off a steady relationship	1. Yes 2. No	
LTEQ-15	You had a serious problem with a close friend, neighbor or relative	1. Yes 2. No	
LTEQ-16	You became unemployed or you were seeking work unsuccessfully for more than 1 month	1. Yes 2. No	
LTEQ-17	You were sacked from your job	1. Yes 2. No	
LTEQ-18	You had a major financial crisis	1. Yes 2. No	
LTEQ-19	You had problems with the police and a court appearance	1. Yes 2. No	
LTEQ-20	Something you valued was lost or stole	1. Yes 2. No	

Section III: Questionnaires to assess social support (SSQ)

S .no	Social Support Questionnaire	Response	Coding
SSQ.21	How many people are so close to you that you can Count on them if you have serious personal problems (choose one option)?	1.None<br>2.1 or 2<br>3.3-5<br>4.More than 5	
SSQ.22	How much concern do people show in what you are doing (choose one option)?	1. No concern and interest<br>2.Little concern and interest<br>3. Uncertain<br>4. some<br>5. a lot	
SSQ.23	How easy is it to get practical help from friends or dorm-mates' if you should need it (choose one option)?	1.Very easy<br>2. Easy<br>3. Possible<br>4. Difficult<br>5. Very difficult	

Section IV: Abuse Assessment Screen (AAS)

S .no	Abuse Assessment Screen Questionnaire	Response	Coding
AASQ-24	Have you ever been emotionally or physically abused by your partner or someone important to you?	1. Yes<br>2. No	
AASQ-25	Within the last year, have you been hit, slapped, kicked or otherwise physically hurt by someone?	1. Yes<br>2. No	
AASQ-26	If yes, by whom?	1. Husband<br>2. Ex-husband<br>3. Boyfriend<br>4. Stranger<br>5. Others(specify)_______<br>Number of times _______	

AASQ-27	Since you have been pregnant, have you been hit, slapped, kicked or otherwise Physically hurt by someone?	1. Yes<br>2. No	
AASQ-28	If yes, by whom?	1. Husband<br>2. Ex-husband<br>3. Boyfriend<br>4. Stranger<br>5. Others (specify)<br>Number of times _______<br>Indicate the area of injury:________	
AASQ-29	Score the most severe incident to the following scale:	1. Threats of abuse, including use of a weapon<br>2. Slapping, pushing; no injuries and/or lasting pain<br>3. Punching, kicking, bruises, cuts and/or continuing pain<br>4. Beaten up, severe contusions, burns, broken bones<br>5. Head, internal, and/or permanent injury<br>6. Use of weapon, wound from weapon	
AASQ-30	Within the past year, has anyone forced you to have sexual activities?	1. Yes<br>2. No	
AASQ-31	If yes, by whom?	1. Husband<br>2. Ex-husband<br>3. Boyfriend<br>4. Stranger<br>5. Others (specify)<br>Number of times _______	
AASQ-32	Are you afraid of your partner or anyone you listed above?	1. Yes<br>2. No	

AASQ-33	Do you want us to reveal this information to: (for those who answered yes)<br>1. The obstetricians looking after you	1. Yes<br>2. No	
AASQ-34	2. The medical social worker for further management	1. Yes<br>2. No	

Section V: Self-Reporting Questionnaire (SRQ)

S .no	Self-Reporting Questionnaire	Response	Coding
SRQ-35	Do you often have headaches?	1. Yes 2. No	
SRQ-36	Is your appetite poor?	1. Yes 2. No	
SRQ-37	Do you sleep badly?	1. Yes 2. No	
SRQ-38	Are you easily frightened?)	1. Yes 2. No	
SRQ-39	Do your hands shake?	1. Yes 2. No	
SRQ-40	Do you feel nervous, tense or worried?	1. Yes 2. No	
SRQ-41	Is your digestion poor?	1. Yes 2. No	
SRQ-42	Do you have trouble thinking clearly?	1. Yes 2. No	
SRQ-43	Do you feel unhappy?	1. Yes 2. No	
SRQ-44	1 Do you cry more than usual?	1. Yes 2. No	
SRQ-45	Do you find it difficult to enjoy your daily activities?	1. Yes 2. No	
SRQ-46	Do you find it difficult to make decisions?	1. Yes 2. No	
SRQ-47	Is your daily work suffering?	1. Yes 2. No	
SRQ-48	Are you unable to play a useful part in life?	1. Yes 2. No	
SRQ-49	Have you lost interest in things?	1. Yes 2. No	
SRQ-50	Do you feel that you are a worthless person?	1. Yes 2. No	
SRQ-51	Has the thought of ending your life been on your mind?	1. Yes 2. No	
SRQ-52	Do you feel tired all the time?	1. Yes 2. No	
SRQ-53	Do you have uncomfortable feelings in your stomach?	1. Yes 2. No	

SRQ-54	Are you easily tired?	1. Yes 2. No	

Section VI: behaviors substance use

S.no	Substance use questionnaire	Response	Coding
SUQ.55	Have you used any kind of alcohol drinks in last 3 months?	1. Yes<br>2. No	
SUQ.56	Have you ever used alcohol drinks in your life?	1.Yes<br>2. No	
SUQ.57	Have you used any kind of tobacco products in the last 3 months?	1. Yes<br>2. No	
SUQ.58	Have you ever used tobacco products?	1.Yes<br>2. No	
SUQ.59	Have you used khat in the last 3 months?	1.Yes<br>2. No	
SUQ.60	Have you ever used khat in your life?	1.Yes<br>2.No	
SUQ.61	Have you used any other substance in the last 3 months? If yes, specify it……….	1.Yes<br>2. No	
SUQ.62	Have you ever used any other substance in your life? If yes, specify it…….	1.Yes<br>2. No	

Section VII: clinical factors questions

S .no	clinical factors Questionnaire	Response	Coding
CQ-63	Do you have a history of mental illness told by mental health professionals	1.Yes<br>2. No	
CQ-64	Are mental illness in the family	1.yes<br>2.No	
CQ-65	Do you have a medical illness told by a doctor?	1. Yes<br>2. No	
CQ-66	If yes for Q-65, which one Of the following co-morbid illnesses do you have?	1. HIV/AIDS<br>2. Diabetic Mellitus<br>3. Hypertension<br>4. Others specify-----	

Section VIII: Maternity and Obstetric related factors

S .no	Maternity and Obstetric related Questionnaire	Response	Coding
ORQ-67	pregnancy intention	1. Planed & wanted<br>2. Unplanned but wanted<br>3. Upland & unwanted	
ORQ-68	Gestational age of pregnancy	1. First trimester<br>2. Second trimester<br>3. Third trimester	
ORQ-69	Gender preference	1. Boy 2. Girl	
ORQ-70	Gravid(number of Pregnancy)	1. One 2. Two to four<br>3.five and above	
ORQ-71	Number of live children	1. 0 2. 1-2 3. ≥3	
ORQ-72	History of past pregnancy complication	1. Yes 2. No	
ORQ-73	Complication with current pregnancy	1. Yes 2. No	
ORQ-74	History of neonatal death	1. Yes 2. No	
ORQ-75	History of stillbirth	1. Yes 2. No	
ORQ-76	History of abortion	1. Yes 2. No	
ORQ-77	History of gynecological operation	1. Yes 2. No	

መለያ ቁ.______________________

3. Amharic version of consent form

3.1 የተሳታፊ መረጃ ሰነድ

የምርምር/የጥናት ርዕስ: በነፍሰ ጡር እናቶች ላይ ብዙ ጊዜ ሊከሰቱ የሚችሉ የአምሮ ችግሮችን የያዙ መጠይቆች 2023 ።

ወጪዉን የሚሸፍን አካል፡ ሚዛን-ቴፒ ዩኒቨርሲቲ

መግቢያ:-ይህ የመረጃ የስምምነት ቅፅ የተዘጋጀው እርዕሶ ታሳተፊ እንዲሆኑ ስለተጋበዙበት በምርምር ቡድኑ የሚካሄደዉን ጥናት በተመለከተ የርዕሶን ፈቃደኝነት ለማወቅ እና በነፍሰ ጡር እናቶች ላይ ብዙ ጊዜ ሊከሰቱ የሚችሉ የአምሮ ችግሮችን እና ተያያዥ ጉዳዮችን ለማጥናት ነው።

ሊደርሰ የሚችል ጉዳት፡ በዚህ ጥናት ተሳታፊ በመሆንዎ ምክንያት የሚደርስ ምንም ዓይነት ጉዳት የለም። መጠይቁን ሞልቶ ለማጠናቀቅ ቢበዛ 30 ደቂቃ ይወስድብዎታል።

ጥቅም:- እርዕሶ በዚህ ጥናት ላይ ተሳታፊ በመሆንዎ በቀጥታ ሊያገኙት የሚችሉት ጥቅም ባይኖርም የእርዕሶ ተሳትፎ በጎንደር ዩኒቨርሲቲ አጠቃላይ ስፔሻላይዝድ ሆስፒታል የቅድመ ወሊድ ተከታታዮች የሚመርጡትን የስነ አምሮ እርዳታ አይነት እና ተያያዥ ጉዳዮችን ለይቶ ለማወቅ ይረዳል።

ጥቅማ ጥቅም፡- በዚህ ጥናት ተሳታፊ በመሆንዎ ምንም ዓይነት ክፍያ አይሰጠዎትም።

ሚስጢራዊነት፡- ለዚህ ጥናት የሚሰበሰበዉ መረጃ የግል ጉዳዩችን ያካተተ በመሆኑ ሚስጢረዊ አንዲሆን ጥንቃቄ ተድርጎበታል። ማንንትዎን የሚገልፅ ነገር ይፋ አይሆንብዎትም፤ በሚስጥር ኮድ ይቀመጣል እንጂ፣ እርዕሶም ስምዎትን እነዲነግሩ አይጠበቅቦትም።

በጥናቱ ያለመሳተፍ ወይም የመቆረጥ መብት:- በዚህ ጥናት ያለመሳተፍ መብትዎ ሙሉ በሙሉ የተጠበቀ ነዉ። ለመጠይቁ በሙሉ ወይም በከፊል መልስ አለመስጠት ይችላሉ እንዲሁም በማንኛዉም በፈለጉት ሰዓት ማንኛዉን መብተዎን ሳያጡ የማቆረጥ ሙሉ መብት አለዎት።

ማግኘት የሚችሉት ሰዎች:- ይህ የምርምር ፕሮጀክት በጎንደር ዩኒቨርሲቲ የምርምርና ሥነ-ምግባር ኮሚቴ ተይቶ የሚጸድቀ ነዉ። የበለጠ መረጃ ለማግኘት የሚፈለጉ ከሆነ በሚከተሉት አደራሻዎች መግኘት ይችላሉ። ማንኛዉን ጥያቄ ቢኖረዎት ከዚህ በታች የተጠቀሱትን ግለሰቦች ማግኘትና መጠያቅ ይችላሉ።

የተመራማሪዎች ስም፡ 1. ክበር ተመስገን ስ.ቁ 0922261345 ኢሜል፡ kibertemesgen12@gmail.com

2. ዶ/ር ብርሀኔ ጌትነት (ፒኤችዲ) ኢሜል፡berhanie.getnet.bg@gmail.com

3. ዶ/ር ቢክሰኝ አስራት (ፒኤችዲ) ኢሜል፡biksegnasrat1@gmail.com

4. አገኘሁ አማረ ኢሜል፡ Agegnehu221@gmail.com

በመጨረሻም በጥናቱ ላይ ለመሳተፍ ተስማምተዋል? 1. አዎ 2. አልተስማማሁም

ከተስማሙ ለተሳትዎ እያመሰገንኩ ወደሚቀጥለዉ ክፍል እንድያልፉ አሳስበለሁ::

3.2 የተሳትፎ ማረጋገጫ

የሰነዱን ይዘት የተረዳሁ ሲሆን የምርምር ፕሮጀክቱንም አላማ ተረድቻለሁ። በዚህ ምርመር ፕሮጀክት ላይም ለመሳተፍ ፍቃደኛ ሆኛለሁ። በማንኛውም ሰዓትም ከጥናቱ ራሴን ለማግለል መብት እንዳለኝ አውቃለሁ።

የተሳታፉ ፊርማ _______________ቀን ______________________
የሱፐርቫይዘር ስም እና ፉርማ ______________________ ቀን ____________
የመረጃ ሰብሳቢ ስም እና ፉርማ ______________________ ቀን ____________

ስለተሳተፉ እናመሰግናለን!

3.3 የ አማረኛ መጠይቆች

ጥያቄዎቹ ስምንት ክፍሎች አሏዋቸዉ። ቢበዛ እስከ 30 ደቅቃ ይፈጃሉ። እበክዎ ሁሉንም መልስ ለመመለስ ይሞክሩ። ለግዜዎት እናመሰግናለን።

መመሪያ፡ የሚከተለትን ጥያቀዎች ምርጫ ያላቸዉን በማክበብ እና ምርጫ የሌላቸዉን በጥያቄዉ መሰረት በክፍት ቦታዉ ላይ ይሙሉ።

ክፍል-1- ማህበራዊ መረጃን በተመለከተ መጠይቅ

ተ.ቁ	መጠይቆች	አማራጭ ምላሽ	ኮዴንግ
SDQ-1	ዕድሜዎ ስንት ነው?	_______በአመት	
SDQ-2	የጋብቻ ሁኔታ	1. ያላገበ 3. አግብቶ የፈታ<br>2. ያገባ 4. የሞተበት	
SDQ-3	ሃይማኖት	1.ኦርቶዶክስ 3. ፕሮቴስታንት<br>2. እስላም 4. ካቶሊክ<br>5.ሌላ፤ ይጥቀሱ…….	
SDQ-4	የትምህርት ደረጃዎ	1.ማንበብምሆነመጻፍአልችልም<br>2.አንደኛደረጃያጠናቀቀ (1-8)<br>3. ሁለተኛደረጃያጠናቅቀ (9-12)<br>4. ዲፕሎማ	

		5. ድግሪ አና ከዚያ በላይ	
SDQ-5	ከሚከተሉት ስራ መደቦች የትኛው እርስዎን ይገልጣል?	1.የቤት እመቤት 4. ነጋዴ<br>2.የመንግስት ተቀጣሪ 5. ሌላ<br>3.የግል ተቀጣሪ	
SDQ-6	የቤተሰብ ብዛት	1. ዜሮ እስከ ሁለት 2.ከሶስት እስከ አራት 3.አምስት እና ከዚያ በላይ	
SDQ-7	ቤት ዉስጥ ዉሳኔ ሰጪ ማን ነው	1. እኔ 2. ባለቤቴ<br>3. በጋራ 4 አማቶቼ	
SDQ-8	አማካይ የወር ገቢዎ ስንት ነው	___________________ብር	

ክፍል-2 መሰረታዊ የህይወት ገጠመኞችን የሚዳስሱ መጠየቆች

ተ.ቁ	መጠይቆች	መልስ	ኮዴንግ
LTEQ-9	በእርስዎ ላይ ከባድ ሕመም ፣ ጉዳት ወይም ዛቻ ደርሶብዎት ያቃል?	1.አዎ 2.የለም	
LTEQ-10	በቅርብ ዘመድዎ ላይ ከባድ ሕመም፣ ጉዳት ወይም ጥቃት የደረሰበት አለ?	1.አዎ 2.የለም	
LTEQ-11	የእርስዎ ወላጅ፣ ልጅ ወይም ባለቤትዎ የሞተአለ?	1.አዎ 2.የለም	
LTEQ-12	የቅርብ የቤተሰብ ጓደኛ ወይም ሌላ ዘመድ (አክስቴ, የአጎት ልጅ, አያት) የሞተ አለ	1.አዎ 2.የለም	
LTEQ-13	በችግር ምክንያት ከትዳርዎ ተለያየተዋል	1.አዎ 2.የለም	
LTEQ-14	የተረጋጋ ግንኙነት አቋርጠሃል	1.አዎ 2.የለም	
LTEQ-15	ከቅርብ ጓደኛ፣ ጎረቤት ወይም ዘመድ ጋር ከባድ ችግር አጋጥሞዎታል	1.አዎ 2.የለም	
LTEQ-16	ሥራ አጥ ሆነው ወይም ሥራ ሲፈልጉ ከ1 ወር በላይ ሳይሳካልዎ ነበር	1.አዎ 2.የለም	
LTEQ-17	ከስራዎ ተባረው ያውቃሉ	1.አዎ 2.የለም	
LTEQ-18	ትልቅ የገንዘብ ችግር ገጥሞዎት ያቃል	1.አዎ 2.የለም	

LTEQ-19	ከፖሊስ እና ከፍርድ ቤት ጋር ችግሮች ነበሩብዎት	1.አዎ 2.የለም	
LTEQ-20	ዋጋ የሰጡት ነገር ጠፍቶዎት ወይም ተሰርቀው ያቃሉ	1.አዎ 2.የለም	

ክፍል-3- የማህበረሰብ ድጋፍ ለመለየት የሚያገለግሉ ጥያቄዎች

ተ.ቁ	የማህበረሰብ ድጋፍ ለመለየት የሚያገለግሉ ጥያቄዎች	መልስ	ኮዴንግ
SSQ.21	ከባድ የግል ችግር ቢያጋጥምዎ በእነሱ ላይ የሚተማመኑባቸዉ ለእርስዎ በጣም ቅርብ የሆኑ ስንት ሰዎች ካጠገብዎ ሊሆኑ ይችላሉ?	1. ማንም<br>2.1ወይም2<br>3.3-5<br>4.6 ወይምከዛበላይ	
SSQ.22	እርስዎ በሚያደርጉት ነገር ውስጥ ሰዎች ምን ያህል ትኩረት ያሳያሉ	1.ምንም<br>2.በጣምጥቂት<br>3.ኣይታወቅም<br>4.የተወሰነ<br>5.በጣምብዙ	
SSQ.23	በፈለጉ ጊዜ ከጓደኛዎ ወይም ከጎረቤትዎ እርዳታ ለማግኘት ለርስዎ ምን ያህል ቀላል ነዉ	1.በጣምቀላልነው<br>2.ቀላልነው<br>3.ማግኘትይቻላል<br>4.ኣስቸጋሪነው<br>5.በጣምኣስቸጋሪነው	

ክፍል 4 ጥቃትን ለመለየት መጠይቅ

ተ.ቁ	ጥያቄዎች	መልስ	ኮዴንግ
AASQ-24	በባልደረባዎ ወይም ለእርስዎ አስፈላጊ በሆነ ሰው በስሜት ወይም በአካል ተበድለው ያውቃሉ?	1.አዎ 2.የለም	
AASQ-25	ባለፈው ዓመት ውስጥ፣ተመተው ወይም የአካል ጉዳት ደርሶብዎታል?	1.አዎ 2.የለም	
AASQ-26	አዎ ከሆነ በማን? (የሚመለከተውን ሁሉ ይክበቡ)	1.ባል<br>2. የቀድሞ ባል<br>3. የወንድ ጓደኛ<br>4. እንግዳ<br>5.ሌሎች<br>(ይግለጹ)________<br>የጊዜ ብዛት________	

AASQ-27	ነፍሰ ጡር ሆነው ተመተው፣ ወይም የአካል ጉዳት ደርሶብዎት ያቃል?	1.አዎ 2.የለም	
AASQ-28	አዎ ከሆነ በማን? (የሚመለከተውን ሁሉ ያክበቡ)	1.ባል<br>2. የቀድሞ ባል<br>3. የወንድ ጓደኛ<br>4. እንግዳ<br>5.ሌሎች<br>(ይግለጹ)________<br>የጊዜ ብዛት_______	
AASQ-29	ከሚከተሉ ውስጥ በጣም ከባድ የሆነውን ክስተት ያክብቡ	1. መሳሪያን ጨምሮ ማስፈራሪያዎች ደርሰውብኛል<br>2. በጥፊ ተመትቸ ውይም ተገፍትሬ አቃለሁ ነገር ግን; ምንም ጉዳት ወይም ዘላቂ ህመም የለም<br>3.ተመትቸ ቆስያለሁ ወይም ህመሙ አለ<br>4. ከባድ ድብደባ ወይም, ቃጠሎ, ደርሶብኛል አጥንቴ እስከመሰበር<br>5. ጭንቅላቴ ላይ ከባድ ጉዳት ደርሶብኛል<br>6. የጦር መሳሪያ በመጠቀም አቁስሎኛል	
AASQ-30	ባለፈው ዓመት ውስጥ፣ የፆታ ግንኙነት እንድትፈጽሙ ያስገደደዎት አለ?	1. አዎ 2. የለም	
AASQ-31	አዎ ከሆነ በማን	1.ባል<br>2. የቀድሞ ባል<br>3. የወንድ ጓደኛ	

		4. እንግዳ<br>5.ሌሎች<br>(ይግለጹ)________<br>የጊዜ ብዛት________	
AASQ-32	ከላይ ከተዘረዘሩት ውስጥ የሚፈሩት ሰው አለ?	1. አዎ 2.የለም	
AASQ-33	ይህንን መረጃ ለሚከተሉት እንድንገልጽ ይፈልጋሉ፡ (አዎ ብለው ለመለሷቸው ጥያቄዎች)<br>1.የማህፀን ሐኪ እንዲያይዎት	1አዎ 2 የለም	
AASQ-34	2.የሕክምና ማህበራዊ ሰራተኛ ለተሻለ አገልግሎት	1.አዎ 2. የለም	

ክፍል 5 የተለመዱ የአዕምሮ ህመሞችን ለመዳሰስ የተዘጋጀ መጠይቅ

ተ.ቁ	ጥያቄዎች	መልስ	ኮዴንግ
SRQ-35	ብዙ ጊዜ ራስ ምታት ያምዎታል?	1.አዎ 2. የለም	
SRQ-36	የምግብ ፍላጎትዎ ቀንሷል?	1.አዎ 2. የለም	
SRQ-37	እንቅልፍ ለመተኛትይ ቸገራሉ?	1.አዎ 2. የለም	
SRQ-38	በቀላሉ የፍርሃት ስሚት ይሰማዎታል?	1.አዎ 2. የለም	
SRQ-39	እጆችዎ ይንቀጠቀጣል?	1.አዎ 2. የለም	
SRQ-40	ፍርሃት ወይም ጭንቀት ስሜት ይሰማዎታል?	1.አዎ 2. የለም	
SRQ-41	ምግብ በሆድዎ ውስጥ ይበረክትብዎታል?	1.አዎ 2. የለም	
SRQ-42	በግልፅ ለማሰብ ይቸገራሉ?	1.አዎ 2. የለም	
SRQ-43	ደስተኛ ያልሆነ ስሜት ይሰማዎታል?	1.አዎ 2. የለም	
SRQ-44	ከወትሮው በተለየ ያለቅሳሉ?	1.አዎ 2. የለም	
SRQ-45	በዕለት ተዕለት እንቅስቃሴዎ መደሰት ይከብደዎታል?	1.አዎ 2. የለም	
SRQ-46	ውሳኔ ለመወሰን ይቸገራሉ?	1.አዎ 2. የለም	
SRQ-47	የዕለት ተዕለት ሥራዎስቃይ የበዛበት ነው?	1.አዎ 2. የለም	
SRQ-48	በህይወት ውስጥ ጠቃሚ ሚና መጫወት አይችሉም?	1.አዎ 2. የለም	
SRQ-49	ለነገሮች ፍላጎት የማጣት ስሜት አለ?	1.አዎ 2. የለም	
SRQ-50	ዋጋ ቢስ ሰው እንደሆኑ ይሰማሃል?	1.አዎ 2. የለም	
SRQ-51	ህይወታችሁን የመጨረስ ሀሳብ በአእምሮዎ ውስጥ ነበር?	1.አዎ 2. የለም	
SRQ-52	ሁል ጊዜ ድካም ይሰማዎታል?	1.አዎ 2. የለም	

SRQ-53	በሆድዎ ውስጥ የማይመቹ ስሜቶች አሉዎት?	1.አዎ 2. የለም	
SRQ-54	በቀላሉ ይደክሞዎታል?	1.አዎ 2. የለም	

ክፍል 6: የኣደንዛዥ ዕፅ ኣጠቃቀም መለያ ጥያቄዎች

ተ.ቁ	ጥያቄዎች	መልስ	ኮዴንግ
SUQ.55	ባለፉት 3 ወራት ዉስጥ ማንኛዉንም አይነት አልኮል ተጠቅመዋል?	1.አዎ 2.የለም	
SUQ.56	በህይወትዎመጠጦችንተጠቅመውያዉቃሉ(ለምሳሌ፡አረቄ፣ጠላ፣ጠጅ. ወዘተ)?	1.አዎ 2. የለም	
SUQ.57	ባለፉት 3 ወራት ዉስጥ ሲጋራ ተጠቅመዋል?	1.አዎ 2. የለም	
SUQ.58	በህይወትዎ ሲጋራ ተጠቅመው ያውቃሉ?	1.አዎ 2. የለም	
SUQ.59	ባለፉት 3 ወራት ዉስጥ ጫትተጠቅመዋል?	1.አዎ 2. የለም	
SUQ.60	በህይወትዎ ጫት ተጠቅመው ያውቃሉ?	1.አዎ 2. የለም	
SUQ.61	ባለፉት 3 ወራት ውስጥ ሌላ ማንኛውንም ንጥረ ነገር ተጠቅመዋል? አዎ ከሆነ ይግለጹ………	1.አዎ 2. የለም	
SUQ.62	በሕይወትዎ ውስጥ ሌላ ማንኛውንም ንጥረ ነገር ተጠቅመው ያውቃሉ? አዎ ከሆነ ይግለጹ…….	1.አዎ 2. የለም	

ክፍል 7.አካላዊና አእምሮአዊ ህመሞችን ለመለየት የሚረዱ ጥያቄዎች

ተ.ቁ	ጥያቄዎች	መልስ	ኮዴንግ
CQ-63	ከዚህ በፊት የታወቀ የአእምሮ ህመም አለዎት	1.አዎ 2. የለም	
CQ-64	በቤተሰብ የታወቀ የኣእምሮ ህመም ኣለ	1.አዎ 2. የለም	
CQ-65	ለረጅም ጊዜ የሚያምዎት አካላዊ ህመም አለ	1.አዎ 2. የለም	
CQ-66	ለQ-65 አዎ ከሆነ፣ ከሚከተሉት አብሮ-ተላላፊ በሽታዎች የትኛው ነው ያለዎት?	1. ኤችአይቪ / ኤድስ<br>2. የስኳር በሽታ<br>3. የደም ግፊት	

		4. ሌላ ካለ ይጥቀሱ---	

ክፍል 8-ከእናትነት እና ከእርግዝና ጋር የተያያዙ ምክንያቶች የያዘ መጠይቅ

ተ.ቁ	ጥያቄዎች	መልስ	ኮዴንግ
ORQ-67	የአሁኑን እርግዝና ከመፀነስሽ በፊት፣ ስለእርግዝናው ምን ታስቢ ነበር?	1. በጊዜው ለመፀነስ ፈልጌ ነበር<br>2. በጊዜው ለመፀነስ አልፈለኩም ነበር፣ አሁን ግን ፈልጌዋለሁ<br>3. በጊዜው ወይንም ከዚያ በኋላ ለመፀነስ አልፈለኩም ነበር	
ORQ-68	የእርግዝና ጊዜዎ	1. የመጀመሪያ አጋማሽ<br>2. ሁለተኛ አጋማሽ<br>3. ሦስተኛው አጋማሽ	
ORQ-69	የጾታ ምርጫ	1.ወንድ 2.ሴት	
ORQ-70	ግራቪድ (ስንተኛ እርግዝናዎ ነው)	1. የመጀመሪያ<br>2. ከሁለት እስከ አራት<br>3.አምስት እና ከዚያ በላይ	
ORQ-71	በህይወት ያሉ ልጆች ቁጥር	1. ዜሮ 2. አንድ ወይም ሁለት<br>3. ሶስት እና ከዚያ በላይ	
ORQ-72	ያለፈው እርግዝና ችግር ነበር	1.አዎ 2. የለም	
ORQ-73	ያለፈው እርግዝና ችግር ነበር	1.አዎ 2. የለም	
ORQ-74	ካሁን በፊት የሕፃን ሞት አጋጥሞሽ ያውቃል?	1.አዎ 2. የለም	
ORQ-75	ካሁን በፊት ሞተው የተወለዱ ህፃናት ነበሩሽ?	1.አዎ 2. የለም	
ORQ-76	አስወርዶሽ ያውቃል?	1.አዎ 2. የለም	
ORQ-77	የመሃጸን ቀዶ ጥገና አድርገው ያቃሉ?	1.አዎ 2. የለም	
